# EEG-Based Recognition of Self-Imagined Emotions Using Multi-Domain Features and Subject-Specific Calibration

**DOI:** 10.3390/s26216858

**Published:** 2026-10-29

**Authors:** Charoenporn Bouyam, Nannaphat Siribunyaphat, Bukhoree Sahoh, Si Thu Aung, Yunyong Punsawad

**Affiliations:** 1 School of Informatics, Walailak University, Thasala 80160, Nakhon Si Thammarat, Thailand; charoenporn.bo@wu.ac.th (C.B.); nannaphat.sir@mail.wu.ac.th (N.S.); bukhoree.sa@wu.ac.th (B.S.); 2 Informatics Innovative Center of Excellence, Walailak University, Thasala 80160, Nakhon Si Thammarat, Thailand; 3 Division of Mathematics and Science, Parami University, Washington, DC 20036, USA; sithuaung1@parami.edu.mm

**Keywords:** brain–computer interface, electroencephalography, emotion recognition, self-imagined emotion, EEG connectivity, subject-specific calibration

## Abstract

Self-imagined emotional imagery, in which individuals internally generate emotional states without external induction, plays a key role in shaping affective brain–computer interface (BCI) systems. Robust generalization across individuals is challenging due to significant variability in EEG patterns. This study evaluates a multi-domain electroencephalography (EEG) decoding framework for self-imagined emotion recognition, integrating time–frequency features, functional connectivity, effective connectivity, and subject-specific calibration. EEG data were collected from 38 participants and categorized into four emotional conditions using the valence–arousal model. Time–frequency features were extracted using the wavelet transform; functional connectivity was assessed using the phase locking value (PLV) and Pearson’s correlation; and effective connectivity was estimated using Granger causality. For comparison, a two-dimensional convolutional neural network (CNN) based on the continuous wavelet transform was implemented. Under pooled sample-wise cross-validation, PLV-based features combined with a support vector machine (SVM) achieved 97.0% accuracy, while the CNN reached 94.75%. Under leave-one-subject-out evaluation, accuracy declined to approximately 25–30%, indicating limited generalization to unseen participants. With subject-specific calibration using 75% of the target participant’s data, the SVM achieved 95.0% accuracy. The results support the recognition of four emotional conditions under the evaluated protocol when data from the target participant are available. However, cross-subject generalizability remains a significant limitation. As the study was conducted under controlled experimental conditions, further research involving independent populations and experimental settings is necessary to evaluate the framework’s robustness and applicability.

## 1. Introduction

Emotions significantly influence human cognition and behavior, shaping perception, decision-making, actions, and overall well-being [1,2]. Emotional disorders represent a substantial proportion of psychiatric conditions, affecting over one billion individuals worldwide through manifestations such as depression, anxiety, and stress-related disorders [3]. The assessment and monitoring of emotional states, particularly in clinical environments such as palliative care, are recognized as essential. Accurate evaluation in these settings supports person-centered care, improves communication, and guides clinical decision-making [4,5]. However, assessing internal emotional states remains challenging, especially for individuals with limited communication abilities [6,7].

Electroencephalography (EEG) [8,9] is a noninvasive, cost-effective technique for recording scalp electrical activity with high temporal resolution. EEG is widely used in brain–computer interface (BCI) systems and healthcare [10,11], as it directly measures neural activity associated with emotional states, unlike behavioral or peripheral signals such as heart rate or skin conductance. This capability allows assessment of internal emotional states that may not be externally observable [12,13]. In clinical palliative care, objective monitoring of emotional states can support personalized treatment and improve communication between patients and caregivers.

Current EEG-based emotion recognition methods often rely on stimulus-driven paradigms, in which images [14], audio [15], or videos [16,17] are used to elicit emotional responses [18]. Although these methods perform well in controlled environments, their effectiveness may decrease in real-world contexts where emotional reactions arise naturally. In self-imagined emotion paradigms, participants generate emotions by imagining them, making this approach more representative of natural emotional experiences and more suitable for clinical applications, particularly for individuals with communication difficulties. Decoding self-imagined emotions from EEG data remains challenging because of low signal-to-noise ratios, variability, and individual differences [19,20], which reduce robustness and generalizability. Developing techniques that can effectively extract meaningful EEG features while addressing individual differences is essential for practical clinical and BCI applications.

Feature extraction in EEG-based emotion recognition often relies on time–frequency analysis. For example, the discrete wavelet transform (DWT) [21,22,23] decomposes EEG signals into time–frequency bands, enabling the extraction of spectral features. However, these features are limited in their ability to capture interactions among brain regions. Consequently, recent studies have focused on connectivity-based representations, which better characterize relationships within brain networks. Functional connectivity measures, such as the phase locking value (PLV) [24,25,26,27], which quantifies synchronization between EEG signals, and Pearson correlation [28,29,30], a statistical measure of linear association, quantify synchronization across EEG channels. Effective connectivity methods, such as Granger causality [31], are statistical techniques for determining directional influences between signals and characterizing causal interactions between brain regions, thereby providing a more comprehensive description of the brain activity underlying emotional conditions.

Deep learning, especially convolutional neural networks (CNNs), is a type of artificial neural network [31,32] that processes data with a grid-like topology and has been widely used for EEG-based emotion recognition to automatically extract spatiotemporal features [33,34,35]. This reduces reliance on manually designed features and improves the ability to learn complex patterns. The models generally require substantial data sets and are prone to overfitting, performing well on training data but poorly on unseen data, especially when data is scarce, which is common in clinical environments [36].

Moreover, inter-individual variability challenges model generalization, as EEG patterns and emotional responses vary across subjects, thereby limiting subject-independent evaluations. Personalization strategies, such as few-shot learning (training or adapting a model with a small number of subject-specific samples), can be adopted to enable model calibration with limited subject-specific data [37,38]. Among these personalization strategies, the present study adopts a calibration-based approach, in which a small quantity of labelled data from the target subject is collected before use, rather than a meta-learning formulation.

## 2. Related Works

Table 1 summarizes representative EEG-based emotion recognition studies across paradigms, feature encodings, and validation strategies. Early approaches, such as those of Gil and Singh [23], rely on single-domain time–frequency features (DWT) and use subject-dependent evaluation. More recent studies, including Cui et al. [24] and Yuvaraj et al. [35], incorporate functional connectivity and spatiotemporal representations within CNN-based frameworks, improving performance; however, these methods largely depend on externally induced stimuli. Although John and Babu [37] introduced few-shot learning for subject-independent evaluation, their approach remains limited to stimulus-driven paradigms. In contrast, Bouyam et al. [39] explored self-imagined emotion recognition, but this work was restricted to single-domain features and subject-dependent validation.

Despite recent advances, several challenges remain in EEG-based self-imagined emotion recognition. First, existing studies frequently rely on individual feature domains, which may provide only a partial representation of the underlying neural dynamics. Second, substantial inter-subject variability can lead to a considerable reduction in classification performance when models are evaluated on unseen participants. Therefore, systematic evaluation of complementary EEG representations, together with subject-specific calibration, remains important for understanding which representations are most discriminative and how much target-subject data may be required to obtain improved performance.

To address these challenges, the present study systematically evaluates multiple EEG feature representations for self-imagined emotion recognition. The investigated representations include time–frequency features, functional connectivity, effective connectivity, and CWT-based deep learning representations. Rather than assuming that these representations can be directly fused, the study compares their discriminative performance under a common evaluation framework and further examines the effect of subject-specific calibration on recognition performance. Additionally, a subject-specific calibration strategy is incorporated to enhance subject-independent performance and mitigate inter-individual variability. The principal contributions are as follows:

1. A systematic evaluation of multiple EEG feature representations for self-imagined emotion recognition, including time–frequency, functional connectivity, effective connectivity, and CWT-based representations.
2. A comparative analysis of connectivity-based and time–frequency representations using conventional machine-learning classifiers.
3. An evaluation of subject-specific calibration using different proportions of target-subject data to examine the effect of inter-subject variability on emotion classification performance.
4. An analysis of subject-independent generalization using LOSO evaluation, highlighting the substantial performance gap between pooled sample-wise evaluation and unseen-subject evaluation.

## 3. Materials and Methods

This section describes the experimental paradigm, EEG acquisition and preprocessing procedures, feature-extraction methods, feature selection, classification models, and evaluation strategies used for self-imagined emotion recognition. Multiple EEG representations were evaluated independently to compare their discriminative characteristics. In addition, subject-specific calibration was investigated using increasing proportions of target-subject data.

**Figure 1:**
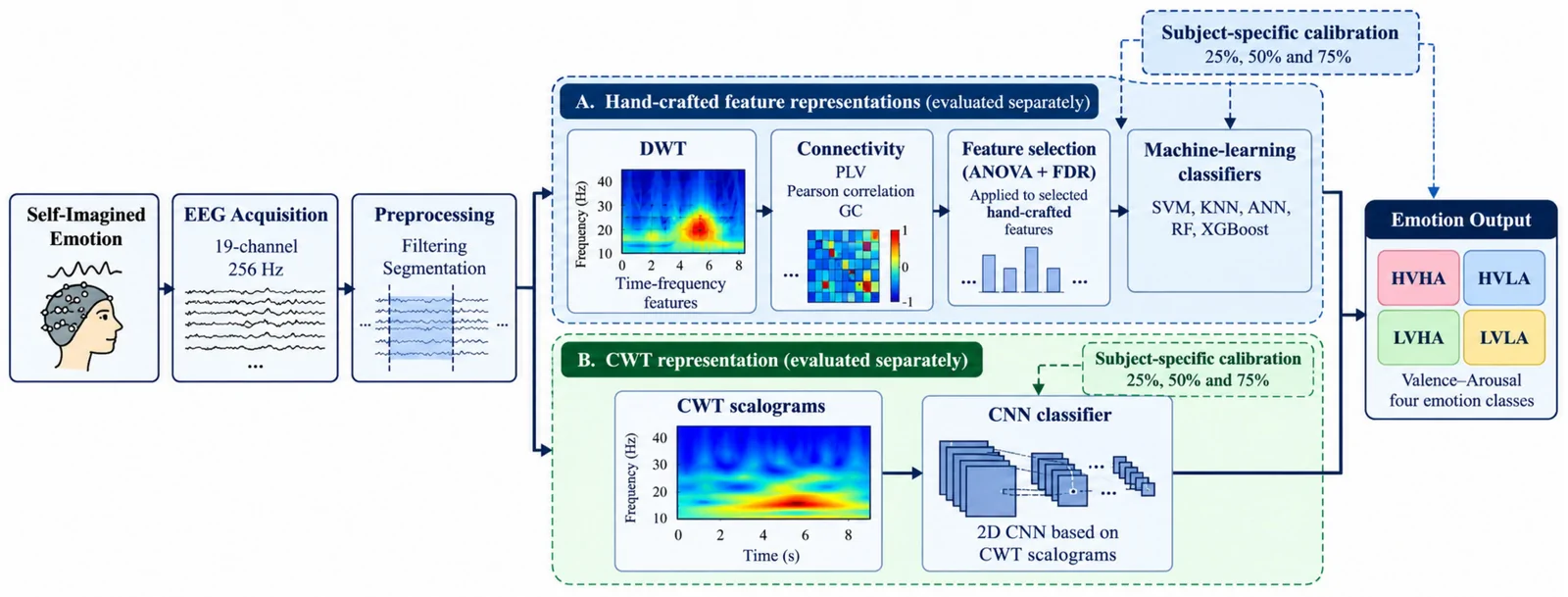
Overview of the proposed multi-domain EEG-based emotion recognition framework incorporating subject-specific calibration.

Figure 1 illustrates the overall system architecture. Following EEG acquisition under the self-imagined emotion paradigm, the signals undergo preprocessing and are analyzed through two separate pathways. The first pathway evaluates hand-crafted feature representations, including discrete wavelet transform (DWT) and connectivity features. ANOVA-based feature selection with false discovery rate (FDR) control is applied to the selected hand-crafted features before classification using machine-learning models. The second pathway transforms EEG signals into continuous wavelet transform (CWT) scalograms, which are classified using a convolutional neural network (CNN) without the ANOVA/FDR feature-selection stage. Subject-specific calibration is evaluated at different proportions of target-subject data within the corresponding classification pathways. The final output comprises four emotion classes defined by the valence–arousal model: high valence–high arousal (HVHA), high valence–low arousal (HVLA), low valence–high arousal (LVHA), and low valence–low arousal (LVLA).

### 3.1. A. Participants and Experimental Paradigm

Thirty-eight healthy volunteers (16 males, 22 females; age 19–23 years, mean 21.29 ± 1.07) participated in this study. All participants reported no history of neurological or psychiatric disorders. The Human Research Ethics Committee of Walailak University approved the study protocol (Project No. WU-EC-IN-2-164-67; Approval No. WUEC-24-164-01; approved on 30 April 2024). Written informed consent was obtained from all participants before the experiment.

To investigate EEG activity associated with self-imagined emotional states, the experimental protocol was based on an extended version of a previously developed self-imagined emotion paradigm [39]. Participants were instructed to recall and engage in personal experiences associated with predefined target emotions. The paradigm included an external facial-expression cue before the imagination period; therefore, the subsequent EEG segment was considered a self-imagination period preceded by an emotion-priming cue rather than a completely stimulus-free paradigm.

**Figure 2:**
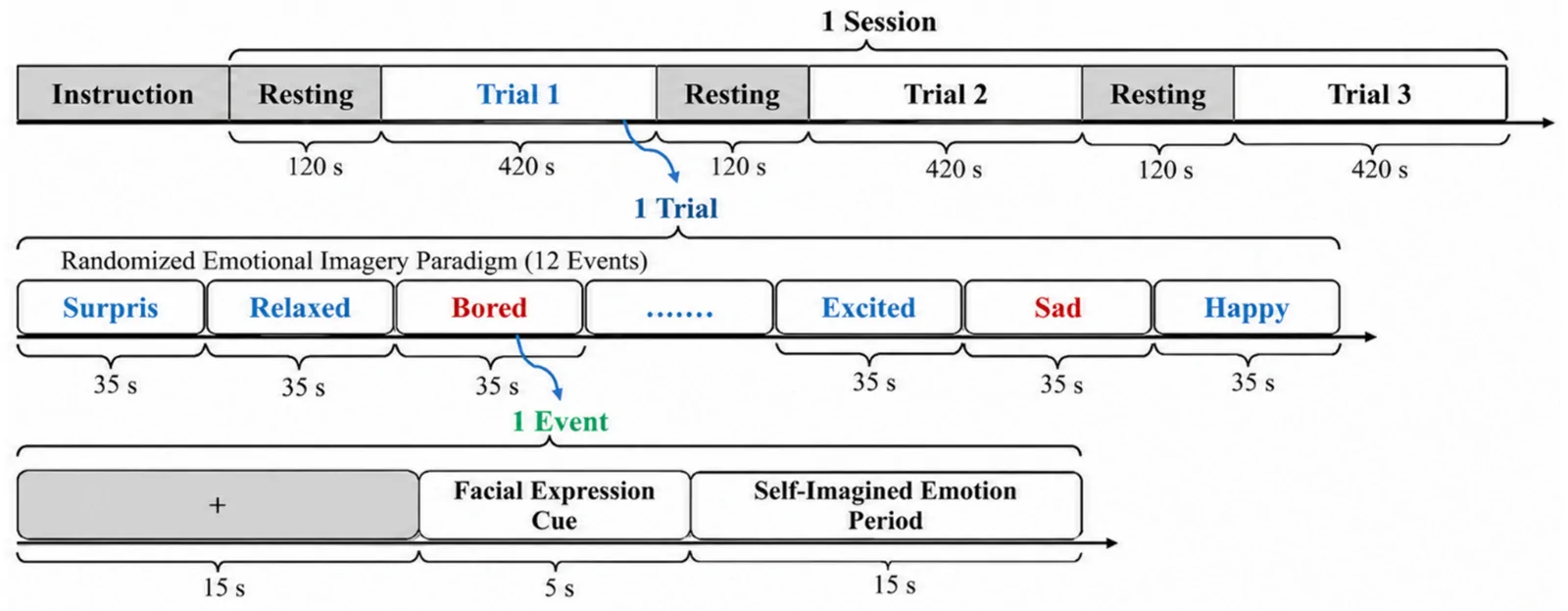
Experimental paradigm for self-imagined emotion: active internal evocation of emotional states.

Twelve target emotions were grouped into four valence–arousal categories based on Russell’s Circumplex Model of Affect, which represents emotional states in a two-dimensional space defined by valence and arousal [41]. By categorizing both dimensions into high and low levels, the emotional states were organized into four classes: high valence–high arousal (HVHA), high valence–low arousal (HVLA), low valence–high arousal (LVHA), and low valence–low arousal (LVLA), as shown in Table 2. This four-class labeling approach has been adopted in previous EEG-based emotion recognition studies [42]. The target emotions were presented in randomized order to reduce potential sequence and carry-over effects. Each emotional event lasted 35 s and consisted of three phases: (1) a 15-s baseline fixation period, (2) a 5-s facial-expression cue used to prime the target emotion, and (3) a 15-s self-imagined emotion period during which participants actively evoked the target emotional state, as illustrated in Figure 2.

The four-class organization was defined according to the valence–arousal framework, with the target emotions assigned to high- or low-valence and high- or low-arousal categories. This grouping was adopted to reduce the classification problem from 12 individual emotion labels to four broader affective states while retaining the two-dimensional structure of emotional experience. Because individual emotions within the same quadrant may still differ in their subjective intensity and semantic characteristics, the four-class formulation should be interpreted as a coarse-grained affective representation rather than evidence that all emotions within a class are psychologically identical. Future studies should further validate the class assignments using participant-level dimensional ratings and examine whether finer-grained emotion labels provide additional discriminative information.

### 3.2. EEG Acquisition and Preprocessing

EEG signals were acquired using the BrainMaster Discovery-24 amplifier (BrainMaster Technologies, Inc., Bedford, OH, USA) in a 19-channel configuration based on the international 10–20 system. All signals were recorded at a sampling frequency of 256 Hz. Initial preprocessing was performed in Discovery software (version 1.6.0) to reduce physiological artifacts and external noise. A 0.5–45 Hz bandpass filter was applied to retain relevant neural frequency bands (theta, alpha, beta, and low gamma) while removing baseline drift and high-frequency interference. A 50 Hz notch filter was also used to suppress power-line interference. After preprocessing, EEG signals from the 15-s emotional imagination phase were segmented using a sliding window approach. A window length of 5 s and a step size of 2.5 s (50% overlap) were used. This approach increases the number of training samples while preserving temporal continuity, allowing finer characterization of emotion-related neural dynamics. Overlapping windows also help retain transient signal features, improving the stability of subsequent feature extraction and classification. This segmentation strategy is commonly used in EEG analysis to balance temporal resolution and statistical reliability.

The 15-s self-imagination segment was divided into 5-s analysis windows with a 2.5-s step, yielding 50% overlap between adjacent windows. This approach increased the number of analysis samples while preserving temporal information. However, overlapping windows share EEG data, and random sample-wise partitioning may place highly similar samples in both training and test sets, potentially inflating performance estimates. Therefore, the pooled cross-validation results reflect sample-wise discrimination rather than generalization to independent trials. Future validation should use trial-level partitioning, assigning all windows from each trial to the same subset.

### 3.3. Multi-Domain Feature Extraction

Traditional spectral methods, such as the Fast Fourier Transform (FFT) and Power Spectral Density (PSD), were initially explored. However, these approaches are limited for non-stationary EEG signals because they lack temporal localization and do not capture inter-regional brain interactions. To address these limitations, a multi-domain feature-extraction framework is proposed that integrates time–frequency analysis, connectivity features, and deep-learning representations to model EEG signals at multiple levels.

First, time–frequency features were extracted using the Discrete Wavelet Transform (DWT) [43,44], which provides multiresolution analysis suitable for non-stationary signals. EEG signals were decomposed into frequency subbands, and statistical features were computed for each subband. The signal energy is defined as follows:

(1) $$E=\sum_{i=1}^{N}x_{i}^{2}$$

where $x_{i}$ denotes the i-th wavelet coefficient and N is the number of coefficients. Shannon entropy was also computed to quantify signal complexity [45]:

(2) $$En=-\sum_{i=1}^{N}p_{i}\log_{2}\left(p_{i}\right)$$

where $p_{i}\;=\frac{x_{i}^{2}}{E}$ denotes the normalized energy distribution. Additional statistical features (standard deviation, skewness, and kurtosis) were also extracted.

Functional connectivity was analyzed using the phase locking value (PLV) and Pearson correlation to characterize relationships between EEG channels [26,46]. PLV quantifies the consistency of phase differences between two signals over time and is defined as

(3) $$PLV=\frac{1}{N}\left|\sum_{t=1}^{N}e^{i\left(\emptyset_{x}\left(t\right)-\emptyset_{y}\left(t\right)\right)}\right|$$

where $\emptyset_{x}\left(t\right)$ and $\emptyset_{y}\left(t\right)$ denote the instantaneous phases of signals x and y at time t, respectively, and N is the number of samples. Before phase extraction, EEG signals were filtered using a fourth-order Butterworth band-pass filter. The instantaneous phase was obtained using the Hilbert transform. The frequency bands were defined as delta (0–4 Hz), theta (4–8 Hz), alpha (8–16 Hz), beta (16–32 Hz), and gamma (32–45 Hz). Pearson correlation was used to quantify the linear association between pairs of EEG signals and is defined as

(4) $$r_{x,y}=\frac{\sum_{t-1}^{N}\left(x\left(t\right)-\bar{x}\right)\left(y\left(t\right)-\bar{y}\right)}{\sqrt{\sum_{t-1}^{n}{\left(x\left(t\right)-\bar{x}\right)}^{2}}\sqrt{\sum_{t-1}^{n}{\left(y\left(t\right)-\bar{y}\right)}^{2}}}$$

where x(t) and y(t) denote the EEG signals at time t, $\bar{x}$ and $\bar{y}$ their mean values, and N is the number of samples.

Effective connectivity was evaluated using bivariate Granger causality (GC) [47] to characterize directed predictive relationships between scalp EEG channels. GC assesses whether the past values of a source signal y improve the prediction of a target signal x beyond the information provided by the target signal’s own past.

For each directed pair from source channel (y) to a target channel (x), (denoted as y→x), restricted and unrestricted linear autoregressive (AR) models were fitted. The restricted model predicted the target signal (x) using only its own past values, yielding residuals $\varepsilon_{2}$. The unrestricted model additionally included the past values of the source signal (y), yielding residuals $\varepsilon_{1}$. The conventional GC measure is defined as

(5) $${GC}_{y\to x}=\ln\left(\frac{var\left(\varepsilon_{2}\right)}{var\left(\varepsilon_{1}\right)}\right)$$

For feature extraction, the F-statistic from the sum-of-squared-residuals (SSR) F-test was utilized instead of the log-variance ratio. This test assesses whether incorporating the source signal’s past values significantly improves prediction accuracy under the joint null hypothesis that all source lag coefficients equal zero. The F-statistic is calculated as

(6) $$F_{y\to x}=\frac{\left({SSR}_{R}-{SSR}_{U}\right)/p}{{SSR}_{U}/\left(N_{eff}-k_{U}\right)}$$

where ${SSR}_{R}$ and ${SSR}_{U}$ denote the residual sums of squares from the restricted and unrestricted models, respectively, evaluated across the same time points. The parameter (p) denotes the number of restricted lag coefficients associated with the source signal (y). $N_{eff}$ represents the effective number of observations per analysis window after accounting for the initial p lag samples. $k_{U}$ is the number of estimated parameters in the unrestricted model ($k_{U}$ = 2p + 1, including an intercept term). The degree-of-freedom for F-distribution are defined by p in the numerator and $N_{eff}-k_{U}$ in the denominator.

The continuous 19-channel EEG recordings were segmented into 5-s sliding windows with 50% overlap (corresponding to a 2.5-s step size). Within each window, bivariate GC analysis was performed across all 342 directed channel pairs (19 × 18), excluding self-connections. The computed F-statistic for each directed pair was retained as an effective connectivity feature.

A fixed autoregressive model order of p = 3 samples was used across all channel pairs, without automatic order optimization via information criteria such as the Akaike information criterion (AIC) or the Bayesian information criterion (BIC). Formal stationarity tests and residual diagnostic assessments were not performed; consequently, the standard linear AR modeling assumptions were not explicitly verified prior to feature extraction.

Finally, the Continuous Wavelet Transform (CWT) was used to generate time–frequency representations suitable for deep learning [48]. CWT produces high-resolution two-dimensional scalograms that preserve spatial, spectral, and temporal information. A convolutional neural network (CNN) was used to automatically extract high-level features from these representations. The CWT is defined as follows:

(7) $$CWT\left(s,t\right)=\frac{1}{\sqrt{\left|s\right|}}\int_{-\infty }^{\infty }x\left(\tau \right)\psi^{*}\left(\frac{\tau -t}{s}\right)d\tau$$

where s is the scale parameter, t is time, τ is the translation variable, and ψ(t) is the mother wavelet function. The resulting scalograms were used as inputs to the CNN to learn high-level spatio-temporal patterns.

These corresponding feature domains capture EEG attributes across multiple levels, including local signal properties (time–frequency), inter-regional interactions (connectivity), and high-level representations (deep learning), thereby improving discrimination of self-imagined emotional conditions.

### 3.4. Feature Selection and Classification

#### 3.4.1. ANOVA-Based Feature Selection Procedure with FDR Correction

The combination of multi-domain features produces a high-dimensional feature space, which can increase computational complexity and the risk of overfitting, thereby limiting cross-subject generalization. To address this, a one-way analysis of variance (ANOVA) F-test was used to assess the discriminative power of each feature across the four emotion classes. ANOVA was chosen for its computational efficiency, interpretability, and ability to identify features with statistically significant class separability.

To control for false positives due to multiple comparisons, the Benjamini–Hochberg false discovery rate (FDR) correction was applied [46,49]. Compared to more conservative corrections (e.g., Bonferroni), FDR offers a balance between sensitivity and statistical reliability, making it appropriate for high-dimensional EEG feature spaces.

As summarized in Table 3, the statistical analysis reports the proportion of features with significant (*p* < 0.05) and highly significant (*p* < 0.001) differences across emotional states. Features were then ranked by their FDR-adjusted *p*-values. To examine the effect of feature dimensionality, subsets of the most informative features (Top 20, 50, 75, and 100) were selected for classification.

**Figure 3:**
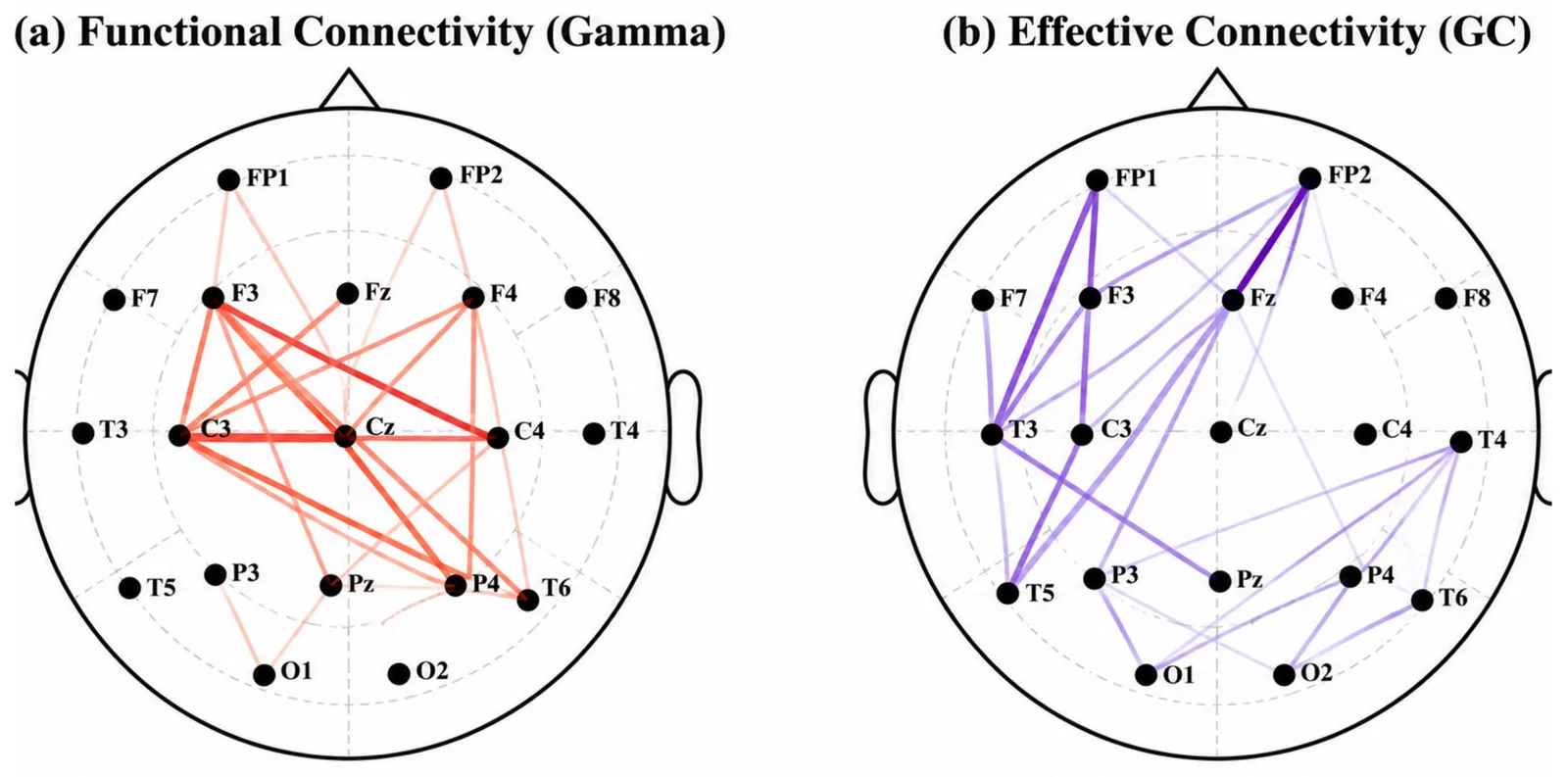
Examples of EEG connectivity features identified using ANOVA F-statistics. Feature selection included all analyzed EEG frequency bands; gamma-band (32–45 Hz) examples are shown here. Nodes represent EEG channels arranged according to the international 10–20 system, and edges represent selected connectivity features. (**a**) Functional connectivity measured using the phase-locking value (PLV). (**b**) Effective connectivity estimated using Granger causality (GC), illustrating directional relationships between EEG channels. Edge color and thickness indicate ANOVA F-statistic values.

The top discriminative features from each domain are listed in Table A1 (Appendix A). In the time–frequency domain, features from the prefrontal region (Fp1), particularly in the beta and gamma bands, demonstrated strong discriminative power. In the connectivity domains, inter-hemispheric functional connections (e.g., C3–C4) and top-down effective connectivity pathways (e.g., Fp2 → Fz) exhibited the highest F-statistics. A visualization of these discriminative connectivity patterns is provided in Figure 3.

Figure 3 presents the most discriminative brain network features identified using ANOVA F-statistics, highlighting connectivity patterns that contribute most to emotion classification. In Figure 3a, functional connectivity (phase-locking value in the gamma band) shows strong inter-hemispheric synchronization, particularly between central regions (e.g., C3–C4) and frontal areas, indicating coordinated activity across distributed brain regions. The prominence of gamma-band synchronization suggests involvement in higher-order cognitive and affective integration. In Figure 3b, effective connectivity (Granger causality) reveals directional information flow, primarily originating from frontal regions (e.g., Fp2 → Fz), reflecting top-down modulation associated with emotional regulation and executive control. Edges with higher F-statistics correspond to features with greater discriminative power. These patterns emphasize the role of frontal and inter-hemispheric networks and support the effectiveness of connectivity-based features for discriminating self-imagined emotional states.

#### 3.4.2. Classification Model and Deep Learning Approach

Building on prior work with traditional classifiers, including support vector machines (SVMs), k-nearest neighbor (KNNs), artificial neural networks (ANNs/MLPs), and random forests (RFs), this study extends the classification system to accommodate multi-domain feature vectors. To further enhance performance on high-dimensional features, XGBoost was incorporated because of its robustness against overfitting and its capacity to approximate complex nonlinear relationships [50].

All selected features were evaluated using these classifiers. Hyperparameters were optimized via grid search with cross-validation on the training data to ensure a fair comparison. The search space is detailed in Appendix B, and the optimal configurations are summarized in Table 4.

In addition to handcrafted feature-based models, a deep learning approach was implemented to learn hierarchical representations directly from data. Specifically, a two-dimensional convolutional neural network (CNN) was applied to continuous wavelet transform (CWT)-based time–frequency representations [48]. The CNN architecture was selected for its capacity to capture spatial and temporal patterns in EEG signals. The network comprises three convolutional blocks with batch normalization, ReLU activation, and max pooling, followed by global average pooling and a fully connected layer with L2 regularization. Dropout was used to reduce overfitting, and training was conducted with the Adam optimizer, incorporating early stopping and learning-rate scheduling. This framework enables a systematic comparison between traditional machine learning models based on handcrafted features and deep learning approaches based on learned representations.

**Figure 4:**
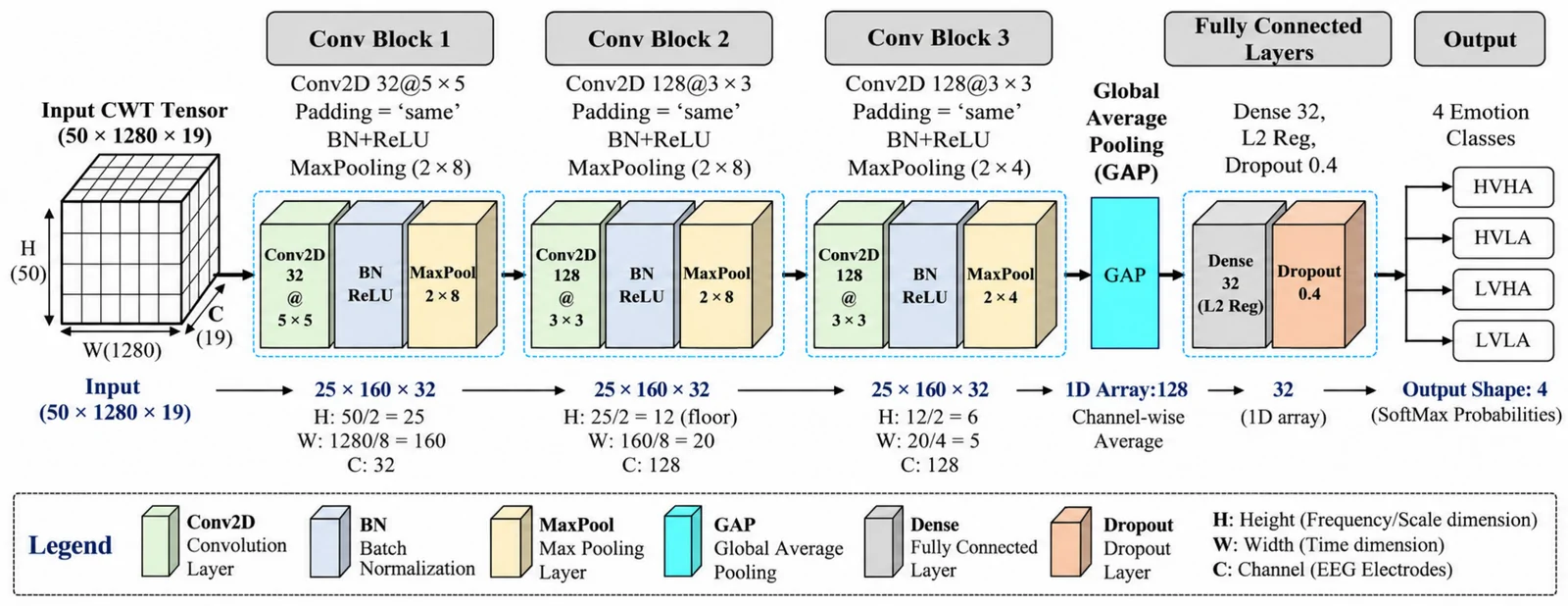
Architecture of the proposed two-dimensional convolutional neural network (2D-CNN) for EEG-based emotion recognition.

The architecture of the proposed 2D-CNN is shown in Figure 4. The model takes CWT-based time–frequency representations as input with dimensions 50 × 1280 × 19. It consists of three convolutional blocks, each containing a Conv2D layer, batch normalization, a ReLU activation, and a max pooling layer, which progressively reduce the feature map dimensions. A global average pooling layer generates a 128-dimensional feature vector, followed by a fully connected layer with dropout (0.4) and L2 regularization. A softmax layer produces probability outputs for the four emotional classes (HVHA, HVLA, LVHA, LVLA).

The CNN training hyperparameters reported in Table 5 were kept unchanged across the evaluation settings. No additional hyperparameter optimization was performed during LOSO evaluation or subject-specific calibration. During subject-specific calibration, the CWT-based CNN was trained using only the designated calibration data from the target subject, while the remaining target-subject data were reserved for testing. This procedure is referred to as target-subject retraining rather than fine-tuning of the LOSO-trained model. The architecture and training hyperparameters remained fixed throughout this procedure.

#### 3.4.3. Validation Strategy and Performance Metrics

Two complementary evaluation strategies were used. First, pooled sample-wise stratified 10-fold cross-validation was performed to evaluate classification performance when samples from multiple participants were included in the same dataset. Because the partitioning was performed at the sample level rather than strictly at the participant or trial level, this evaluation should not be interpreted as a measure of subject-independent generalization. Furthermore, the use of overlapping EEG windows may result in highly similar samples being distributed across different folds. Therefore, the results obtained from this evaluation should be interpreted as within-dataset classification performance rather than as a definitive estimate of generalization to unseen trials or participants.

Second, leave-one-subject-out (LOSO) cross-validation was performed to evaluate classification performance on participants excluded from model training [18,51]. In each LOSO iteration, data from one participant were held out as the target subject, while data from the remaining participants were used for model development. This evaluation was used to examine the extent to which the evaluated feature representations and classification models generalized across participants.

Subject-specific calibration was subsequently evaluated within the LOSO framework using 25%, 50%, or 75% of the target subject’s data for retraining, with the remaining samples used for testing. These calibration levels represent different proportions of target-subject data used for model adaptation. Because the test subsets may differ across calibration levels, the results should not be interpreted as a controlled comparison using a fixed test set.

Feature ranking was performed using one-way analysis of variance (ANOVA) with Benjamini–Hochberg false discovery rate (FDR) correction, followed by selection of the highest-ranked features. In the original analysis, this feature-ranking procedure was performed prior to the cross-validation evaluation. Consequently, evaluation data may have influenced feature selection, potentially inflating the estimated classification performance. Therefore, the reported cross-validation results are not based on a fully nested feature-selection evaluation and should be interpreted in light of this limitation.

Classification performance was evaluated using the metrics reported in the corresponding results tables. Results from pooled sample-wise cross-validation, LOSO evaluation, and subject-specific calibration were interpreted separately because these protocols assess sample-wise discrimination, performance on unseen subjects without calibration, and performance after target-subject calibration, respectively.

## 4. Results

### 4.1. Multi-Domain Feature Extraction

To assess the proposed multi-domain feature-extraction framework, subject-dependent performance was evaluated using 10-fold stratified cross-validation. Both handcrafted features (time–frequency and connectivity-based) and deep learning representations (CWT) were examined.

#### 4.1.1. Feature Selection Using ANOVA with FDR Correction

**Figure 5:**
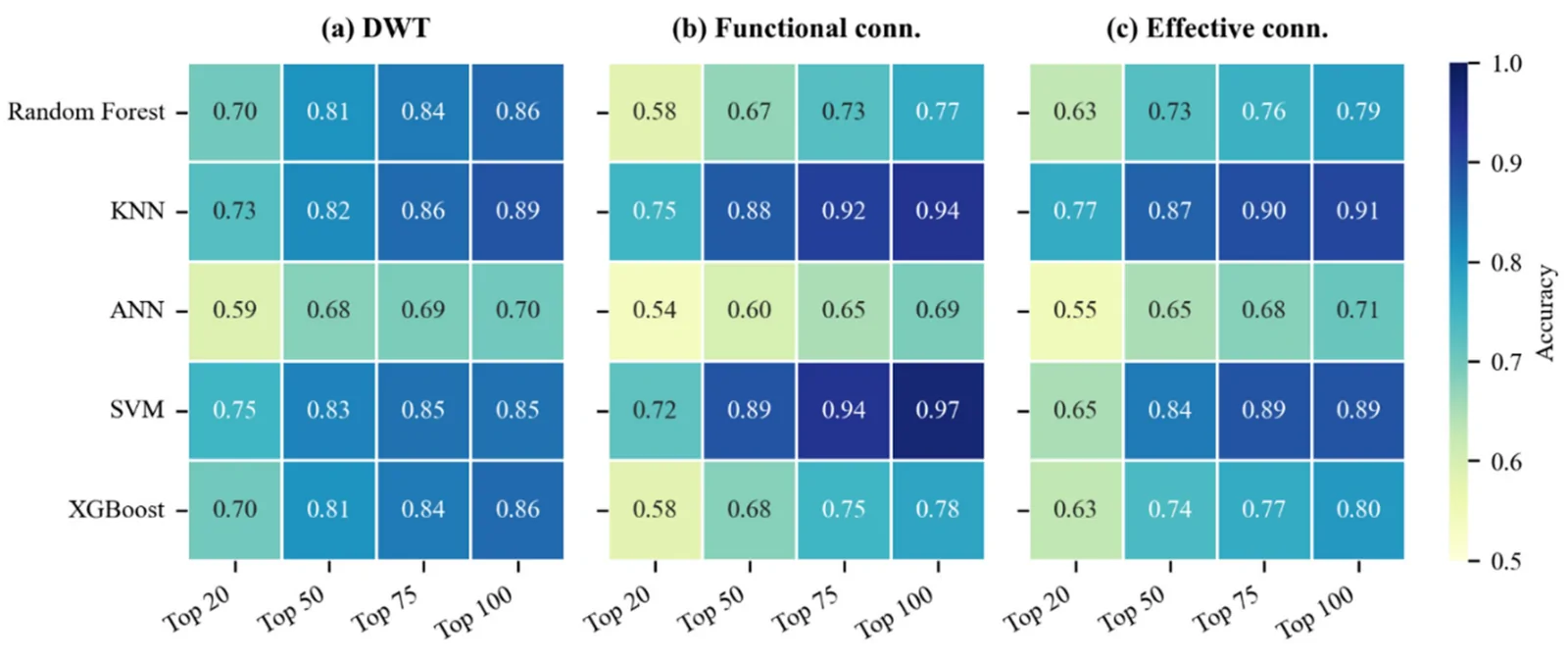
Classification accuracy heatmaps across feature dimensionality and domains: (**a**) time–frequency features (DWT), (**b**) functional connectivity (PLV and Pearson Correlation), and (**c**) effective connectivity (Granger causality). Results are presented for the top 20, 50, 75, and 100 selected features across multiple classifiers. Color intensity represents accuracy.

Classification results across feature domains are shown in Figure 5. Accuracy increases as the number of selected features rises from Top 20 to Top 100, indicating that higher-dimensional subsets capture more discriminative information. However, performance plateaus beyond Top 75, suggesting that selecting 75–100 features offers a favorable balance between accuracy and computational cost.

In the time–frequency domain (Figure 5a), KNN achieves the highest accuracy (0.89 ± 0.01) with the Top 100 features, followed by XGBoost and random forest (approximately 0.86). In contrast, connectivity-based features yield superior performance. Functional connectivity (Figure 5b) achieves the highest accuracy, with SVM reaching 0.97 and KNN reaching 0.94. Effective connectivity (Figure 5c) also performs well, with KNN achieving 0.91 and SVM 0.89.

Overall, connectivity-based features outperform time–frequency features, likely because they capture inter-regional interactions. The strong performance of SVM further indicates that margin-based classifiers are effective for high-dimensional connectivity representations.

#### 4.1.2. Performance of Deep Learning Models Using CWT

The performance of the proposed 2D-CNN using CWT representations is summarized in Table 6. Under pooled sample-wise cross-validation performance, the model achieved an overall classification accuracy of 94.75%, with precision, recall, and F1-scores of 0.95 across all emotion classes. These results indicate that the CNN can effectively learn spatiotemporal patterns directly from time–frequency representations without explicit feature selection. However, this performance was obtained under subject-dependent conditions and does not directly reflect cross-subject generalization.

### 4.2. Subject-Independent Emotion Recognition (LOSO)

To evaluate generalization to unseen subjects, a leave-one-subject-out (LOSO) cross-validation strategy was used with the optimal feature subset identified through pooled sample-wise cross-validation analysis (Top 100 features). The results are presented in Table 7.

Performance declined substantially across all feature domains and models, with accuracy consistently dropping to approximately 0.25–0.30. This decline reflects the effect of inter-individual variability in EEG signals, which introduces significant domain shifts that conventional training approaches cannot effectively address. Among the evaluated methods, connectivity-based features demonstrated slightly greater stability than time–frequency features, although overall performance remained limited. These findings indicate that models trained solely on pooled multi-subject data are insufficient for practical BCI applications, highlighting the need for subject-calibration strategies.

### 4.3. Impact of Subject-Specific Calibration

Table 7 shows that classification performance consistently improves as the proportion of subject-specific data increases from 25% to 75% across all feature domains. This trend suggests that incorporating even a small amount of target-subject data effectively reduces inter-individual variability. Connectivity-based features exhibit the most substantial improvement, with functional connectivity combined with SVM achieving the highest performance (0.95 ± 0.03) at 75% adaptation. Effective connectivity and time–frequency features also show notable gains, with KNN-based models demonstrating stable improvements. In contrast, the CWT-based CNN shows more limited improvement, indicating greater sensitivity to subject variability.

**Figure 6:**
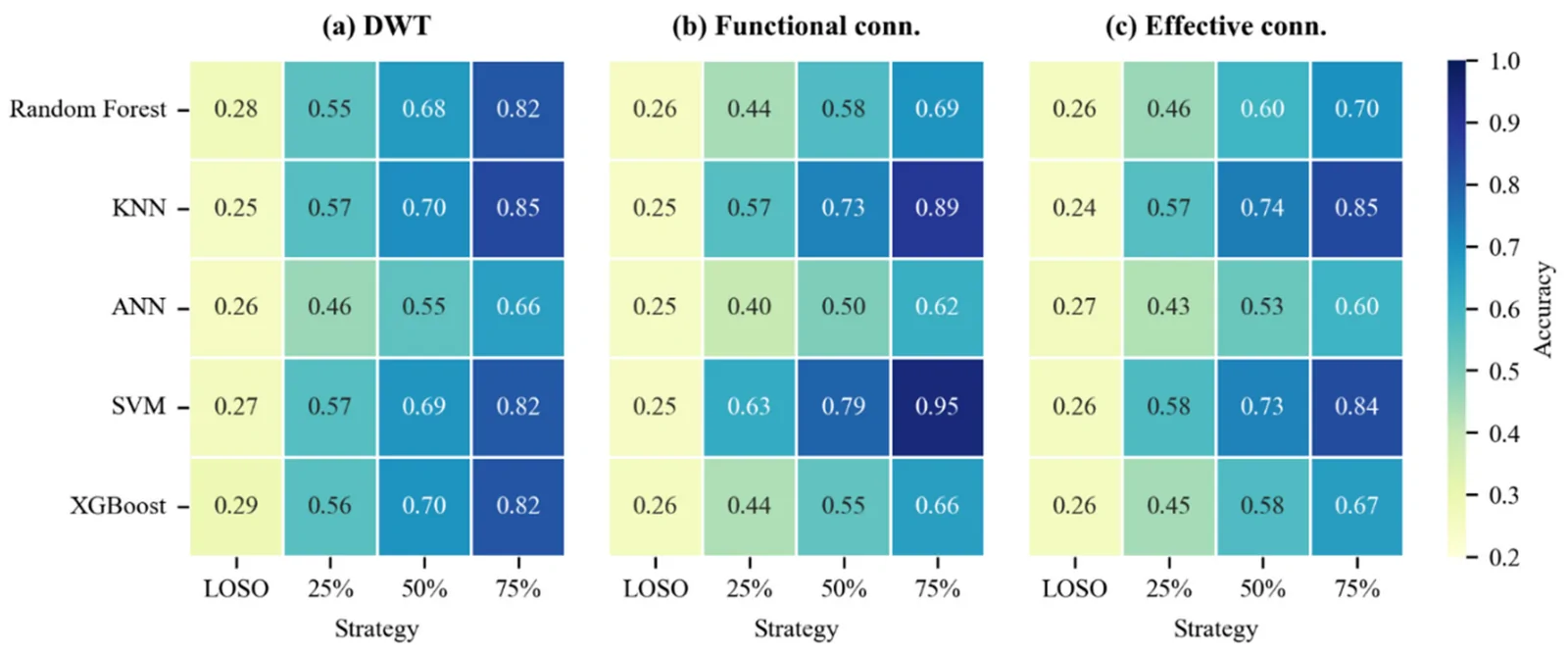
Classification accuracy heatmaps across EEG feature domains and subject calibration settings: (**a**) time–frequency features (DWT), (**b**) functional connectivity, and (**c**) effective connectivity. Results are presented for LOSO and subject-specific calibration (25%, 50%, and 75%). Color intensity represents accuracy.

Figure 6 shows classification performance across feature domains under LOSO and subject-specific calibration settings. Under LOSO evaluation, accuracy remains low (approximately 0.25–0.30), indicating limited cross-subject generalization. Across models and feature domains, accuracy generally increases as the proportion of subject-specific calibration data rises from 25% to 75%, with the largest gains observed for connectivity-based features. Functional connectivity combined with SVM achieves the highest accuracy (approximately 0.95 at 75% calibration), followed by effective connectivity and time–frequency features. In contrast, the evaluated deep learning models show smaller gains across calibration settings. Overall, subject-specific calibration is associated with higher classification accuracy, particularly when combined with connectivity-based representations. However, the 25%, 50%, and 75% calibration settings use different test subsets; therefore, the observed performance differences may reflect both the amount of calibration data and the composition of the test sets. These findings should thus be interpreted as descriptive comparisons rather than controlled comparisons using a fixed test set, and the observed gains cannot be attributed solely to increasing the amount of calibration data.

**Figure 7:**
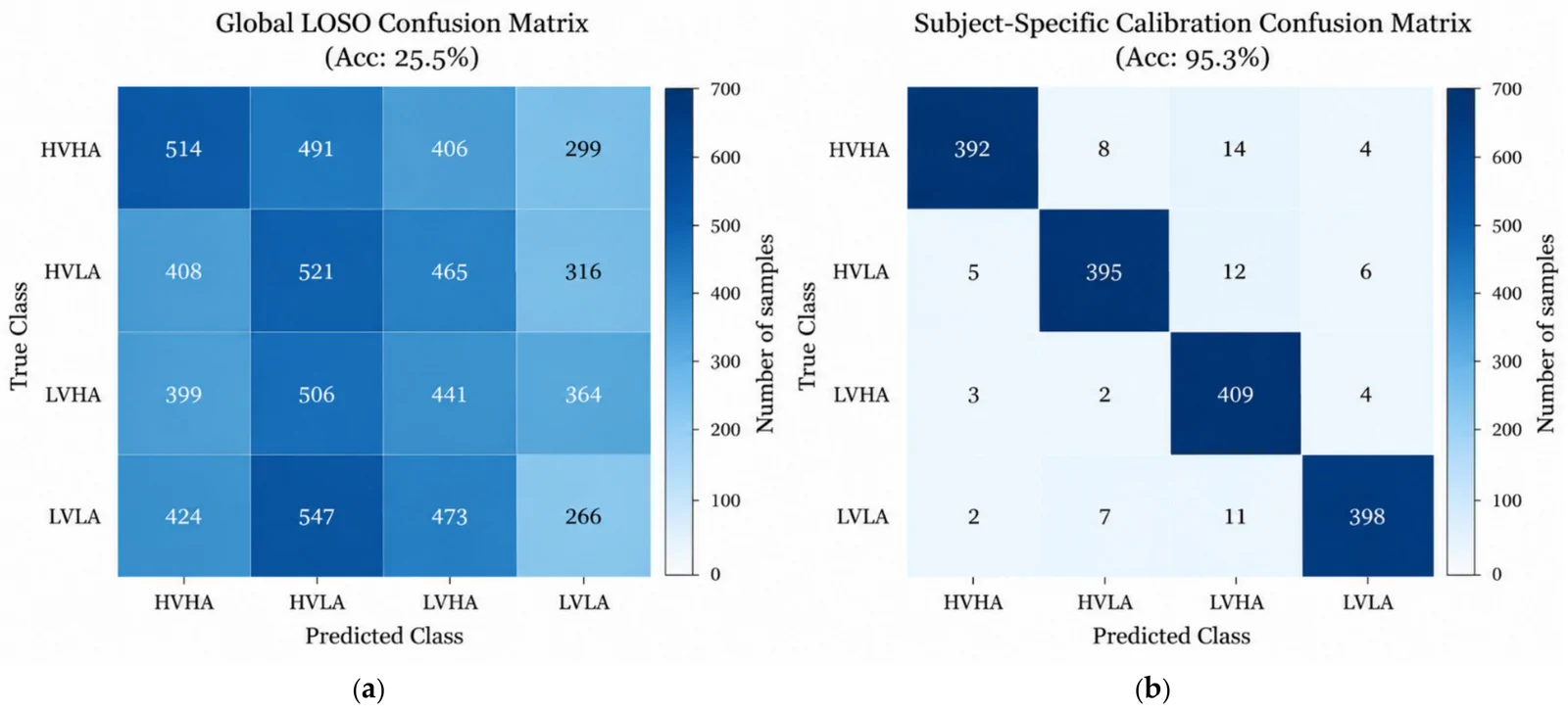
Confusion matrices for the PLV–SVM classifier: (**a**) subject-independent evaluation with leave-one-subject-out (LOSO) cross-validation; (**b**) subject-specific calibration with 75% of the target subject’s data.

To further examine class-wise classification performance, Figure 7 presents the confusion matrices for the PLV–SVM classifier under subject-independent evaluation and subject-specific calibration. Figure 7a shows the LOSO results, with an overall accuracy of 25.46% calculated from the pooled confusion matrix, close to the 25% chance level for four-class classification. The off-diagonal entries indicate substantial misclassification across the four valence–arousal classes, highlighting the difficulty of generalizing to unseen subjects. In contrast, Figure 7b shows results obtained using 75% of the target subject’s data for calibration, yielding a pooled overall accuracy of 95.33%. The much stronger diagonal pattern indicates improved discrimination among the four classes after subject-specific calibration. These confusion matrices provide a class-level illustration of the performance difference between subject-independent evaluation and calibration, complementing the aggregate accuracy results reported in Table 7.

### 4.4. Comparison of Handcrafted and Deep Learning Approaches

The CWT-based CNN achieved 94.75% accuracy in subject-dependent evaluation but showed limited cross-subject generalization in LOSO evaluation. Table 8 summarizes its classification accuracy under LOSO and subject-specific calibration settings, along with statistical comparisons between each calibration level and LOSO.

Statistical comparisons between LOSO and each subject-specific calibration level were based on paired accuracy values from the same participants. The normality of the paired differences was assessed using the Shapiro–Wilk test. A paired *t*-test was used when the normality assumption was met; otherwise, the Wilcoxon signed-rank test was applied. The resulting *p*-values were adjusted across the three comparisons using the Holm method. Effect sizes were calculated using Cohen’s *d_z_*, defined as the mean within-subject accuracy difference between each calibration setting and LOSO divided by the standard deviation of these paired differences.

Under LOSO evaluation, the CWT-based CNN achieved an accuracy of 0.28 ± 0.04, indicating limited cross-subject generalization. Accuracy increased to 0.31 ± 0.07, 0.45 ± 0.14, and 0.60 ± 0.20 with 25%, 50%, and 75% subject-specific calibration, respectively (Table 8). Compared with LOSO, accuracy was significantly higher at 25% calibration (Holm-adjusted *p* = 0.041) and at both 50% and 75% calibration (Holm-adjusted *p* < 0.001). The corresponding Cohen’s *d_z_* values were 0.47, 1.45, and 1.54, respectively. Handcrafted features, particularly functional connectivity, achieved higher accuracy than the evaluated deep learning approaches across LOSO and subject-specific calibration settings. These findings suggest that connectivity-based representations combined with subject-specific calibration are promising for EEG emotion recognition. However, because the calibration settings used different test subsets, the observed performance differences may reflect both the quantity of calibration data and test-set composition and cannot be attributed solely to the amount of calibration data.

## 5. Discussion

The results offer important insights into EEG-based emotion recognition from both computational and neurophysiological perspectives. Connectivity-based features consistently outperformed time–frequency features, indicating that emotional processing involves interactions across distributed brain networks rather than isolated regions [52,53]. Functional connectivity, in particular, captures synchronization patterns associated with coordinated neural activity during affective processing [54].

The substantial decline in performance under subject-independent (LOSO) conditions highlights the influence of inter-subject variability. Differences in brain structure, cognitive strategies, and emotional responses can cause significant domain shifts among individuals [55,56]. These findings indicate that models trained solely on pooled data are unlikely to generalize effectively without some degree of personalization.

The subject-specific calibration strategy partially addresses this limitation by incorporating a small amount of subject-specific data. Performance improved consistently with more adaptation data, showing that even limited calibration can enhance model accuracy. This is especially relevant in practical settings where minimizing data collection time is important.

The CWT-based CNN achieved strong performance under subject-dependent conditions but showed limited generalization across subjects. This suggests that deep learning models may be sensitive to inter-subject variability when training data are limited [57,58]. In contrast, connectivity-based features demonstrated greater stability across subjects. However, the improvements observed with subject-specific calibration indicate that combining learned representations with subject-specific calibration could be a promising direction for future research.

From a neurophysiological perspective, emotional processing involves coordinated activity across multiple brain regions, including frontal and temporal areas [52,59]. Connectivity features capture these inter-regional interactions, whereas time–frequency features primarily describe local signal characteristics [60]. This distinction may account for the stronger performance of connectivity-based approaches observed in this study.

Several limitations should be noted. The relatively small dataset may limit generalizability, particularly for deep learning models. The controlled experimental setup may not fully reflect real-world emotional responses. Additionally, connectivity analysis was restricted to predefined measures and frequency bands, which may not capture more complex or dynamic interactions. Although the subject-specific calibration strategy improves performance, it still requires subject-specific data and introduces an additional calibration stage, which may increase the computational and operational burden of practical deployment. In particular, connectivity-based feature extraction may require additional processing of newly acquired EEG data during calibration. The present study therefore does not yet establish the computational efficiency or real-time feasibility of the calibration process. Noise, artifacts, and electrode variability were also not extensively investigated and may affect real-world performance.

In practical applications, the proposed framework may support emotion monitoring in settings where users intentionally generate or regulate emotional states. It may also warrant future investigation in individuals with communication or motor impairments. However, these potential applications remain exploratory and require validation beyond controlled laboratory conditions. Future clinical and real-world studies should consider diverse populations and practical factors such as movement-related artifacts, electrode-placement variability, physiological variability, and session-to-session changes.

Future work should focus on improving generalization and usability by collecting larger and more diverse data sets, developing efficient subject-specific adaptation strategies, and reducing the amount of calibration data required. Real-time implementation should also be investigated by evaluating calibration time, computational latency, feature-extraction and connectivity-computation costs, and classification stability. Cross-dataset validation using appropriate public EEG emotion datasets and independently collected datasets would further help assess the robustness and generalizability of the proposed framework across different experimental conditions.

## 6. Conclusions

This study evaluated a multi-domain EEG framework for self-imagined emotion recognition that incorporated time–frequency features, functional and effective connectivity, and subject-specific calibration. Connectivity-based features achieved higher classification accuracy than time–frequency features under pooled sample-wise cross-validation. However, leave-one-subject-out evaluation showed limited generalization to unseen participants. Subject-specific calibration achieved an SVM accuracy of 95.0% using 75% of the target participant’s data, indicating substantial reliance on participant-specific calibration data. These findings should be interpreted in light of the controlled experimental context and the study’s methodological limitations. ANOVA-based feature selection with false discovery rate (FDR) correction was performed before cross-validation, which may introduce information leakage and lead to overly optimistic performance estimates. The reported results therefore require confirmation using an evaluation procedure in which feature selection and model tuning are restricted to the training folds. Clinical utility and real-world applicability remain unverified. Future research should employ fully nested cross-validation, reduce calibration requirements, improve cross-subject generalization, and evaluate robustness using independent datasets. Validation in clinical populations and real-world settings is necessary before considering corresponding applications.

## Figures and Tables

**Table 1:** Comparison of the proposed multi-domain EEG-based emotion recognition framework with existing methods.

Study	Paradigm	Subjects	Features	Method	Validation	Subject Calibration	Accuracy (%)
Gil and Singh [23]	External Stimuli	32	Single-domain (DWT)	Traditional ML	Sub-Dependent	None	80.0–85.0
Cui et al. [24]	External Stimuli	15–32	Functional Connectivity (PLV)	CNN	Sub-Dependent	None	85.0–89.0
Yuvaraj et al. [35]	External Stimuli	32	Spatio-Temporal	3D-CNN + Ensemble	Zero-Shot	None	88.0–92.0
John and Babu [37]	External Stimuli	15–45	Prototype Embeddings	Deep Learning	Sub-Independent	Few-Shot	85.0–90.0
Bouyam et al. [39]	Self-Imagined	20	Single-domain (Statistical)	ML (SVM, KNN, RF, XGBoost)	Sub-Dependent	None	86.0
Pandilova et al. [40]	External Stimuli	32	Multi-domain qEEG (spectral, nonlinear, and connectivity features)	Traditional ML (LR, SVM, RF, XGBoost)	Subject-Independent (LOPO, GroupKFold); Cross-Dataset	Personalized modeling and target-subject calibration evaluated	49.3% (valence), 44.7% (arousal)
Present study	Self-imagined emotion	38	DWT; Functional Connectivity; Effective Connectivity; CWT	SVM, KNN, RF, ANN, XGBoost; CNN	Pooled sample-wise stratified 10-fold CV; Leave-One-Subject-Out (LOSO)	Yes (25%, 50%, and 75% of target-subject data)	Pooled CV: 94–97%; LOSO: 25–30% Calibration: 60–95%

**Table 2:** Categorization of Twelve Target Emotions According to Valence–Arousal Classes.

Valence–Arousal Class	Target Emotions
HVHA (High Valence, High Arousal)	Happy, Excited, Surprised
HVLA (High Valence, Low Arousal)	Pleased, Relaxed, Calm
LVHA (Low Valence, High Arousal)	Angry, Fearful, Disgusted
LVLA (Low Valence, Low Arousal)	Sad, Depressed, Bored

**Table 3:** Statistical summary of EEG feature domains based on one-way ANOVA with false discovery rate (FDR) correction.

Domain	Feature	Total	Significant (*p* < 0.05)		Highly Significant (*p* < 0.001)	
			Amount	%	Amount	%
(DWT)	Energy	95	65	68.4	31	32.6
	SD	95	75	79.0	50	52.6
	Skewness	95	30	31.6	3	3.2
	Kurtosis	95	40	42.1	12	12.6
	Entropy	95	0	0.0	0	0.0
Functional Conn.	PLV	687	263	38.3	96	14.0
	Pearson	171	66	38.6	5	2.9
Effective Conn.	Granger	642	228	35.5	92	14.3

**Table 4:** Optimal hyperparameter configurations for machine learning models across EEG feature domains.

Model	Parameter	Time–Frequency (DWT)	Functional & Effective Connectivity
XGBoost	n_estimators	1000	1000
	learning_rate	0.01	0.01
	max_depth	15	15
	min_child_weight	1	1
	gamma	0	0
	subsample	0.8	0.8
	colsample_bytree	0.7	0.6
	n_jobs	-	−1
KNN	metric	manhattan	manhattan
	n_neighbors	3	3
	weights	distance	distance
SVM	C	10	10
	gamma	0.1	0.1
	kernel	rbf	rbf
	probability	True	True
Random Forest	max_depth	None	None
	min_samples_split	2	5
	n_estimators	300	300
ANN	activation	tanh	relu
	alpha	0.0001	0.01
	hidden_layer_sizes	(100, 50)	(100)
	max_iter	2000	2000

**Table 5:** Training hyperparameters of the CWT-based 2D-CNN.

Hyperparameter	Configuration
Optimizer	Adam
Initial learning rate	(1 × 10^−4^)
Batch size	8
Maximum epochs	30
Early stopping patience	15 epochs
LR reduction factor	0.5
LR scheduler patience	3 epochs

**Table 6:** Performance of the proposed 2D-CNN using continuous wavelet transform (CWT) representations under pooled sample-wise stratified 10-fold cross-validation.

Emotion Class	Precision	Recall	F1-Score
HVHA	0.96	0.95	0.96
HVLA	0.95	0.94	0.95
LVHA	0.94	0.94	0.94
LVLA	0.94	0.96	0.95
Macro Average	0.95	0.95	0.95

**Table 7:** Best-Performing models across EEG feature domains under different subject calibration settings (Mean ± S.D.).

Domain	Model	Strategy			
		LOSO	25% Calibration	50% Calibration	75% Calibration
Time–Frequency (DWT)	KNN	0.25 ± 0.06	0.57 ± 0.06	0.70 ± 0.05	0.85 ± 0.05
Functional Connectivity (PLV)	SVM	0.26 ± 0.05	0.64 ± 0.07	0.80 ± 0.05	0.95 ± 0.03
Effective Connectivity (GC)	KNN	0.25 ± 0.04	0.56 ± 0.05	0.74 ± 0.05	0.85 ± 0.04
Deep Learning (CWT–CNN)	CNN	0.28 ± 0.04	0.31 ± 0.07	0.46 ± 0.14	0.60 ± 0.20

**Table 8:** Classification accuracy and statistical comparisons of the CWT-based CNN model under LOSO and subject-specific calibration settings.

Strategy	Accuracy (Mean ± Std)	Holm-Adjusted *p*-Value (vs. LOSO)	Effect Size (Cohen’s *d_z_*)
LOSO	0.28 ± 0.04	–	–
25% calibration	0.31 ± 0.07	0.041	0.47
50% calibration	0.45 ± 0.14	<0.001	1.45
75% calibration	0.60 ± 0.20	<0.001	1.54

Note: Accuracy is reported as the mean ± standard deviation across participants. All *p*-values compare the corresponding calibration setting with LOSO and were adjusted using the Holm method. Cohen’s *d_z_* represents the mean paired accuracy difference divided by the standard deviation of these differences; positive values indicate higher accuracy under subject-specific calibration. The en dash (–) indicates that the comparison is not applicable because LOSO serves as the reference condition.

## Data Availability

The data presented in this study are available upon request from the corresponding author.

## Appendix A

**Table A1:** Top ten most discriminative eeg features across different domains identified by anova with false discovery rate (fdr) correction.

Domain	Feature	F-Value	*p*-Value	p (FDR)
Time–Frequency (DWT)	Fp1 (Beta-En)	59.70	4.27 × 10^−38^	2.03 × 10^−35^
	Fp1 (Beta-Std)	55.27	2.68 × 10^−35^	6.36 × 10^−33^
	Fp1 (Gamma-Std)	49.50	1.21 × 10^−31^	1.44 × 10^−29^
	Fp1 (Gamma-En)	48.33	6.67 × 10^−31^	6.33 × 10^−29^
	O2 (Gamma-Std)	46.40	1.11 × 10^−29^	7.51 × 10^−28^
	O2 (Gamma-En)	39.80	1.71 × 10^−25^	9.04 × 10^−24^
	T6 (Gamma-Std)	37.35	6.15 × 10^−24^	2.43 × 10^−22^
	Fp1 (Theta-Std)	29.38	7.24 × 10^−19^	2.65 × 10^−17^
	F3 (Theta-Std)	26.58	4.36 × 10^−17^	1.29 × 10^−15^
	T6 (Gamma-En)	25.58	1.89 × 10^−16^	4.99 × 10^−15^
Functional Conn. (PLV)	C3_C4 (Gamma)	27.04	2.24 × 10^−17^	1.92 × 10^−14^
	F3_C4 (Gamma)	24.54	8.65 × 10^−16^	3.70 × 10^−13^
	F3_P4 (Gamma)	19.41	1.58 × 10^−12^	4.50 × 10^−10^
	C3_T6 (Gamma)	18.53	5.73 × 10^−12^	1.22 × 10^−09^
	C3_Cz (Gamma)	18.35	7.49 × 10^−12^	1.28 × 10^−09^
	C3_Fz (Gamma)	17.79	1.69 × 10^−11^	2.41 × 10^−09^
	F3_T6 (Gamma)	17.65	2.07 × 10^−11^	2.53 × 10^−09^
	F3_Cz (Gamma)	17.28	3.56 × 10^−11^	3.80 × 10^−09^
	C3_F4 (Gamma)	16.85	6.69 × 10^−11^	6.00 × 10^−09^
	C3_P4 (Gamma)	16.82	7.01 × 10^−11^	6.00 × 10^−09^
Effective Conn. (GC)	Fp2 → Fz	22.45	1.81 × 10^−14^	6.19 × 10^−12^
	T5 → Fz	17.08	4.67 × 10^−11^	5.50 × 10^−09^
	Fp1 → T3	17.06	4.83 × 10^−11^	5.50 × 10^−09^
	T5 → Fp1	16.63	9.01 × 10^−11^	7.70 × 10^−09^
	Fp2 → T3	16.23	1.62 × 10^−10^	1.11 × 10^−08^
	F7 → T3	15.91	2.59 × 10^−10^	1.48 × 10^−08^
	Pz → T3	15.43	5.26 × 10^−10^	2.57 × 10^−08^
	T6 → O1	14.86	1.21 × 10^−09^	5.17 × 10^−08^
	Fz → P3	14.15	3.41 × 10^−09^	1.30 × 10^−07^
	Fz → T5	12.85	2.27 × 10^−08^	7.77 × 10^−07^

## Appendix B

**Table A2:** Hyperparameter search space for machine learning models used in EEG-based emotion recognition.

Model	Parameter	Search Range
SVM	C	{0.1, 1, 10, 100}
	gamma	{0.001, 0.01, 0.1, 1}
	kernel	{RBF}
KNN	n_neighbors	{3, 5, 7, 9}
	metric	{euclidean, manhattan}
	weights	{uniform, distance}
RF	n_estimators	{100, 200, 300, 500}
	max_depth	{None, 10, 20, 30}
	min_samples_split	{2, 5, 10}
XGBoost	n_estimators	{300, 500, 1000}
	learning_rate	{0.01, 0.05, 0.1}
	max_depth	{5, 10, 15}
	min_child_weight	{1, 3, 5}
	subsample	{0.7, 0.8, 1.0}
	colsample_bytree	{0.6, 0.7, 0.8}
ANN (MLP)	hidden_layer_sizes	{(50,), (100,), (100,50)}
	activation	{tanh, relu}
	alpha	{0.0001, 0.001, 0.01}
	max_iter	{1000, 2000}
